# Clinical utility of long cap-assisted suction: two cases of food bolus and intraprocedural clot removal

**DOI:** 10.1055/a-2695-4427

**Published:** 2025-09-30

**Authors:** Nobutaka Doba, Kosuke Shibayama, Shinzo Abe, Daiki Sakuma, Masanobu Someya, Kazuto Komatsu, Shin Maeda

**Affiliations:** 1 36998 Department of Gastroenterology, Yokosuka City Hospital, Yokosuka, Japan; 2 Department of Gastroenterology, Yokohama City University Graduate School of Medicine, Yokohama, Japan

Cap-assisted suction techniques have been described for food bolus extraction and foreign body removal 1 2 3 . However, video-based documentation of long cap-assisted suction – particularly for piecemeal removal or intraprocedural clot clearance – remains limited ( Fig. 1 ). Herein, we present two cases highlighting the versatility of this approach.

**Fig. 1:**
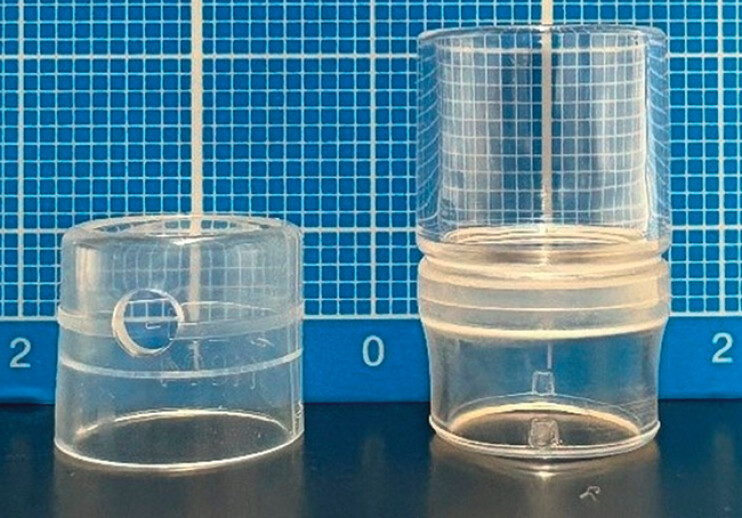
Left: Normal cap (M-201-11804; outer diameter 12.1 mm; tip protrusion length 4 mm; Olympus, Tokyo, Japan). Right: Long cap (MH-463; outer diameter 13.5 mm; tip protrusion length 12 mm; Olympus).

Case 1: A 93-year-old woman presented with complete esophageal obstruction caused by a large food bolus ( Fig. 2 **a** ). The push technique was not feasible ( Fig. 2 **b** ), and retrieval net attempts failed due to poor visualization and a narrowed lumen. Thus, the cap-assisted suction technique was attempted. First, a long transparent cap and overtube were mounted onto the endoscope ( Fig. 2 **c** ). Portions of the bolus were then suctioned into the cap, and the scope was withdrawn and rinsed ( Video 1 ). This cycle was repeated until 141 g of food was removed over 35 minutes under intravenous sedation. No complications occurred during the procedure ( Fig. 2 **d** ).

**Fig. 2:**
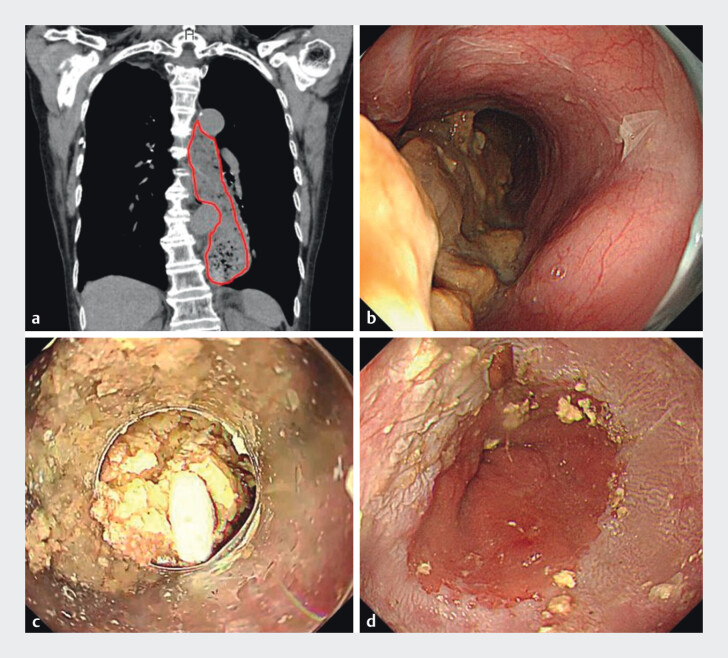
Case 1 images. **a** Pretreatment computed tomography image showing suspected esophageal food bolus impaction. The area delineated by the red line indicates the food bolus. **b** Pretreatment endoscopic image showing a large food bolus in the upper thoracic esophagus; endoscope insertion into the distal esophagus was not possible. **c** Pre-suction endoscopic image: the opening of the long cap positioned at the oral side of the food bolus. **d** Post-treatment endoscopic image showing the esophagogastric junction after complete removal of the food bolus.

Case 2: A 52-year-old man undergoing gastric endoscopic submucosal dissection (ESD) experienced spurting hemorrhage ( Fig. 3 **a, b** ). After achieving hemostasis, a large volume of clotted blood accumulated. Both the retrieval net and short cap-assisted suction techniques were ineffective, and long cap-assisted suction was subsequently attempted ( Video 1 ). The latter technique enabled effective clot removal, allowing safe continuation of ESD ( Fig. 3 **c, d** ).

Clinical utility of long cap-assisted suction.Video 1

**Fig. 3:**
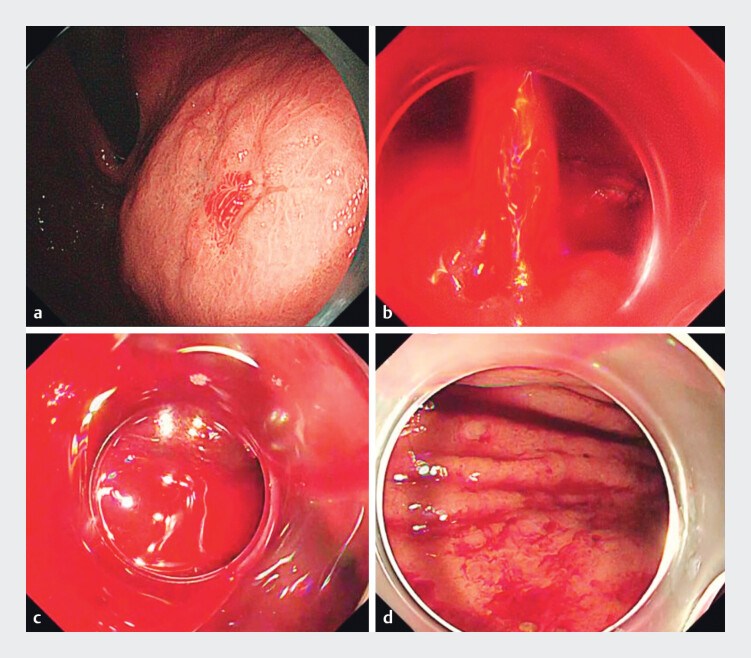
Case 2 images. **a** Endoscopic image before endoscopic submucosal dissection showing early gastric cancer (0–IIc) on the anterior wall of the upper gastric body. **b** Intraprocedural endoscopic image showing active spurting hemorrhage during mucosal incision. **c** Pre-suction endoscopic image demonstrating the long cap opening located adjacent to the clot. **d** Post-suction endoscopic image following clot removal.

Compared with conventional pull-based retrieval or external suction methods 4 5 , long cap-assisted suction offers improved control, soft tissue engagement, and a simplified setup without the need for additional tubing or general anesthesia. It is a simple and reproducible procedure that requires only standard equipment.

This method is particularly advantageous in emergency settings or during therapeutic procedures when time and visibility are critical. It offers a safe, cost-effective solution for difficult bolus or clot removal and may be integrated into routine endoscopy practice.

Endoscopy_UCTN_Code_TTT_1AO_2AD Endoscopy_UCTN_Code_TTT_1AO_2AL

## Notes

Endoscopy E-Videos https://eref.thieme.de/e-videos
*E-Videos* is an open access online section of the journal *Endoscopy* , reporting on interesting cases and new techniques in gastroenterological endoscopy. All papers include a high-quality video and are published with a Creative Commons CC-BY license. Endoscopy E-Videos qualify for HINARI discounts and waivers and eligibility is automatically checked during the submission process. We grant 100% waivers to articles whose corresponding authors are based in Group A countries and 50% waivers to those who are based in Group B countries as classified by Research4Life (see: https://www.research4life.org/access/eligibility/ ). This section has its own submission website at https://mc.manuscriptcentral.com/e-videos .
